# Supplementary material for: The Drosophila HNF4 nuclear receptor promotes glucose-stimulated insulin secretion and mitochondrial function in adults
Source: eLife. 2016 May 17;5:e11183. doi: 10.7554/eLife.11183 (PMC4869932; doi:10.7554/eLife.11183)
Supplement: Supplementary file 2. — The top 10-16 GO categories for each gene set are listed in order of significance along with the number of genes affected in that category, the total number of genes in that category (in parentheses), and the statistical significance of the match. DOI: http://dx.doi.org/10.7554/eLife.11183.020 [file elife-11183-supp2.docx]

**Supplementary Table 2. Top gene ontology categories for the 500 most down- and up-regulated genes in *dHNF4* mutant adults**

**Down-regulated genes (top 500) GO:0016491**

| **GO category** | **Number of genes (total)** | **P value** |
| --- | --- | --- |
| Oxidoreductase activity | 71 (541) | 3.1e-41 |
| Monocarboxylic acid metabolic process | 17 (53) | 6.7e-11 |
| Fatty acid metabolic process | 14 (35) | 2.1e-10 |
| Electron carrier activity | 21 (166) | 4.4e-10 |
| Hydrolase activity | 89 (1550) | 5.2e-09 |
| Developmental process | 20 (2243) | 1.0e-08 |
| Carboxylesterase activity | 21 (122) | 1.4e-08 |
| Lipid metabolic process | 21 (125) | 1.9e-08 |
| Oxidoreductase activity acting on CH-OH donors, NAD or NADP acceptor | 17 (79) | 1.9e-08 |
| Triacylglycerol lipase activity | 12 (37) | 5.3e-08 |
| Biopolymer metabolic process | 10 (1657) | 6.7e-08 |
| Vitamin binding | 9 (18) | 6.7e-08 |
| Fatty acid oxidation | 6 (6) | 6.7e-08 |
| Oxidoreductase activity acting on CH-OH donors | 19 (112) | 7.3e-08 |
| Cellular component organization and biogenesis | 17 (1962) | 7.8e-08 |
| Lipase activity | 16 (78) | 7.8e-08 |

**Up-regulated genes (top 500)**

| **GO category** | **Number of genes (total)** | **P value** |
| --- | --- | --- |
| Immune system process | 34 (202) | 5.5e-31 |
| Multi-organism process | 33 (212) | 4.9e-27 |
| Response to other organism | 32 (131) | 2.5e-19 |
| Response to biotic stimulus | 32 (134) | 4.0e-19 |
| Response to bacterium | 24 (71) | 5.4e-18 |
| Immune response | 32 (147) | 5.5e-18 |
| Antimicrobial humoral response | 26 (89) | 6.5e-18 |
| Defense response | 32 (164) | 1.4e-16 |
| Humoral immune response | 26 (102) | 2.3e-16 |
| Response to stimulus | 70 (982) | 2.8e-16 |

Determined using GOstat: <http://gostat.wehi.edu.au/cgi-bin/goStat.pl>
